# Prolyl-4-hydroxylase α subunit 2 promotes breast cancer progression and metastasis by regulating collagen deposition

**DOI:** 10.1186/1471-2407-14-1

**Published:** 2014-01-02

**Authors:** Gaofeng Xiong, Lei Deng, Jieqing Zhu, Piotr G Rychahou, Ren Xu

**Affiliations:** 1 Markey Cancer Center, University of Kentucky, Lexington, KY 40536, USA; 2 Department of Surgery, University of Kentucky, Lexington, KY 40536, USA; 3 Department of Molecular and Biomedical Pharmacology, University of Kentucky, 741 S. Limestone, BBSRB, Lexington, KY 40536, USA

**Keywords:** Tumor microenvironment, Breast cancer, Collagen deposition, Cancer progression, Cell proliferation

## Abstract

**Background:**

Increased collagen deposition provides physical and biochemical signals to support tumor growth and invasion during breast cancer development. Therefore, inhibition of collagen synthesis and deposition has been considered a strategy to suppress breast cancer progression. Collagen prolyl-4-hydroxylase α subunit 2 (P4HA2), an enzyme hydroxylating proline residues in -X-Pro-Gly- sequences, is a potential therapeutic target for the disorders associated with increased collagen deposition. However, expression and function of P4HA2 in breast cancer progression are not well investigated.

**Methods:**

Gene co-expression analysis was performed in the published microarray datasets to identify potential regulators of collagen I, III, and IV in human breast cancer tissue. Expression of P4HA2 was silenced by shRNAs, and its activity was inhibited by 1, 4-DPCA, a prolyl-4-hydroxylase inhibitor. Three-dimensional culture assay was used to analyze roles of P4HA2 in regulating malignant phenotypes of breast cancer cells. Reduced deposition of collagen I and IV was detected by Western blotting and immunofluorescence. Control and P4HA2-silenced breast cancer cells were injected into fat pad and tail vein of SCID mice to examine effect of P4HA2 on tumor growth and lung metastasis.

**Results:**

Using gene co-expression analysis, we showed that *P4HA2* was associated with expression of *Col1A1*, *Col3A1*, and *Col4A1* during breast cancer development and progression. *P4HA2* mRNA levels were significantly upregulated in breast cancer compared to normal mammary tissue. Increased mRNA levels of *P4HA2* correlated with poor clinical outcome in breast cancer patients, which is independent of estrogen receptor status. Silencing P4HA2 expression or treatment with the P4HA inhibitor significantly inhibited cell proliferation and suppressed aggressive phenotypes of breast cancer cells in 3D culture, accompanied by reduced deposition of collagen I and IV. We also found that knockdown of P4HA2 inhibited mammary tumor growth and metastasis to lungs in xenograft models.

**Conclusion:**

These results suggest the critical role of P4HA2 in breast cancer progression and identify P4HA2 as a potential therapeutic target and biomarker for breast cancer progression.

## Background

Extracellular matrix (ECM) is an important component of tumor microenvironment and plays critical roles in cancer development [1-3]. Collagens are the major structural ECM proteins and form fibers or networks in tumor tissue [4-6]. Cell-collagen interaction controls a variety of cellular activities including proliferation, migration, and invasion through integrin and discoidin domain receptor [7-9]. Enhanced expression and deposition of collagens are associated with tumor development and progression [10-12]. Recent studies demonstrate that increased collagen deposition and crosslinking enhance the stiffness and density of mammary tissue [5,10,13], which is an important risk factor for breast cancer development. Type I collagen has been identified as a prognosis marker and is associated with cancer recurrence in human breast cancer patients [14]. Collagen VI knockout mice have reduced primary tumor formation and growth [12], while enhancing collagen deposition and inhibiting collagen degradation significantly enhances tumor initiation and tumor growth [5,10]. In addition, cancer cell invasion usually occurs at tumor-stromal interfaces with oriented collagen fibers, and aligned collagen fibers can facilitate cell migration and metastasis [5,10,11,15]. These results indicate that increased collagen expression and deposition promotes breast cancer development and progression by enhancing tumor growth and invasion. Therefore, inhibiting collagen synthesis or deposition is a promising strategy to suppress breast cancer progression.

Collagen biosynthesis is a multistep process that involves several post-transcription modification enzymes, and one of the most important members of these enzymes is collagen prolyl-4-hydroxylase [16]. It catalyzes the formation of 4-hydroxyproline by hydroxylating proline residues in -X-Pro-Gly- sequences [17-20]. Collagen prolyl-4-hydroxylase resides within the lumen of the endoplasmic reticulum (ER) [18] and its expression is used as a marker for collagen synthesis [21,22]. Because 4-hydroxyproline residues formed in this reaction are essential for triple helix formation and stabilization of collagen [22-24], inhibiting the prolyl-4-hydroxylases activity efficiently blocks collagen synthesis and deposition. All known vertebrate collagen prolyl-4-hydroxylases are α2β2 tetramers consisting of two α subunits and two β subunits. Each α subunit contains the peptide substrate binding domain and the two catalytic sites of the enzyme, and the β subunits have been identified as protein disulfide isomerases [17,19,25]. Three types of collagen prolyl-4-hydroxylases α isoforms (P4HA1, P4HA2 and P4HA3) have been identified in human tissue. P4HA1 is expressed in most cell types; P4HA2 is mainly expressed in chondrocytes, osteoblasts, and capillary endothelial cells; P4HA3 expression is detected in adult and fetal tissues at very low levels compared to P4HA1 and P4HA2 [18,26]. Increased P4HA2 expression has been detected in many solid tumors, including oral cavity squamous cell carcinoma, papillary thyroid cancer, and breast cancer [27-30], however, the function of P4HA2 in cancer progression largely remains to be determined.

Here, we showed that expression of *P4HA2* and collagen genes (*Col1A1, Col3A1, and Col4A1*) is significantly correlated during breast cancer development and progression, and that increased mRNA levels of *P4HA2* are associated with poor prognosis in breast cancer patients. Silencing P4HA2 or treatment with the P4HA inhibitor attenuates cell proliferation and suppresses aggressive 3D phenotypes, tumor growth, and cancer metastasis, which are accompanied by reduced collagen deposition. These results suggest that P4HA2 promotes breast cancer progression by enhancing collagen deposition and it may serve as a potential therapeutic target for breast cancer.

## Methods

### Antibodies and reagents

The Click-iT® EdU Alexa Fluor® 488 Imaging Kit and Alexa Fluor® 594 phalloidin were from Invitrogen. Matrigel (lrECM) and Type I collagen were from BD Bioscience. ShP4HA2 plasmids were purchased from Sigma. 1, 4-DPCA was purchased from Cayman Chemical. Masson’s trichrome stain kit was purchased from Polysciences, Inc. The following antibodies were obtained as indicated: integrin α6 (Millipore); collagen I (Abcam); collagen IV (Abcam); P4HA2 (Santa Cruz); tubulin (Millipore).

### Cell culture and virus preparation

HMT-3522 T4-2 cells (a kind gift from Dr. Mina J. Bissell) were maintained on tissue culture plastic as previously described [31]. MDA-MB-231 cells were propagated in DMEM/F12 (Sigma) with 10% fetal bovine serum (Invitrogen). MDA-MB-157 cells and ZR-75-1 cells were propagated in DMEM (Sigma) with 10% fetal bovine serum. ZR-75-1 cells: ER-positive and PR positive; T4-2 cells, MDA-MB-231 cells and MDA-MB-157 cells: ER-negative and PR negative.

3D laminin-rich extracellular matrix (3D lrECM) on-top cultures were prepared by trypsinization of cells from tissue culture plastic, seeding of single cells on top of a thin gel of Engelbreth-Holm-Swarm (EHS) tumor extract (Matrigel: BD Biosciences, 354230), and addition of medium containing 5% EHS. T4-2 cells were seeded at a density of 2.1 × 10^4^ cells per cm^2^; MDA-MB-157 cells, ZR-75-1 cells, and MDA-MB-231 cells were seeded at 1.4 × 10^4^ cells per cm^2^. T4-2 cells were maintained in their propagation medium with media change every 2 days. MDA-MB-157 cells, ZR-75-1 cells and MDA-MB-231 cells were maintained in H14 medium with 1% fetal bovine serum. The cell colonies cultured in 3D were imaged and used for immunofluorescence staining at Day 4 after seeding.

HEK293 FT cells were transfected with scrambled RNA sh-control vector or sh-P4HA2-1 (CCGG**GCCGAATTCTTCACCTCTATT**CTCGAG**AATAGAGGTGAAGAATTCGGC**TTTTG), sh-P4HA2-2 (CCGG**GCAGTCTCTGAAAGAGTACAT**CTCGAG**ATGTACTCTTTCAGAGACTGC**TTTTTG) plus packaging lentivector using lipofectamine (Invitrogen). Cancer cells were infected with lentivirus and selected by puromycin 48 h after infection.

### Immunofluorescence and Masson’s trichrome staining

Cells in lrECM gel were smeared on slides, dried briefly, and fixed with 4% paraformaldehyde and permeabilized with 0.5% Triton X-100. Immunostaining was performed as previous described [32]. Stained samples were imaged with a Nikon upright epifluorescence microscope or a confocal system comprised of an Olympus IX81 microscope.

Xenograft tumor sections were de-paraffined and hydrated from xylene, 100% ethanol, 95% ethanol, 85% ethanol and 70% ethanol to distilled water. For Masson’s trichrome staining, slides were re-fixed with Bouin’s solution at 60°C for 60 minutes. Slides were washed in running tap water for 5 minutes and stained in Weigert’s working hematoxyin for 10 minutes. Then they were washed in running tap water for 5 minutes and stained in Biebrich scarlet-acid fuchsin solution for 5 minutes. Slides were rinsed in distilled water and differentiated in phosphomolybdic-phosphotungstic acid solution for 10 minutes, transferred to aniline blue solution and stain for 5 minutes. Slides were rinsed in distilled water and images were taken with a Nikon microscope. The percentage of collagen was quantified by calculating the ratio of blue staining (collagen) area in the total area of the tumor section using Imagescope analysis software [33].

### Western blot analysis

Cells grown on plastic were lysed *in situ* in 2% SDS in PBS buffer containing phosphatase and protease inhibitor cocktails (Calbiochem). Protein concentration was measured using DC™ protein assay (Bio-Rad). Control and shP4HA2 cells were trypsinized and counted; equal amounts of conditional medium (normalized to cell number) were precipitated by pre-cooled acetone. Equal amounts of protein lysates and cell conditional medium were subjected to SDS gel electrophoresis, immunoblotted, and detected with an ECL system (Pierce). Western blotting results were quantified using AlphaInnotech analysis software.

### Transwell invasion assay

The Transwells (Corning) were coated with 60 mL 1 mg/mL Matrigel and incubated for 30 minutes at 37°C. Sh-control or sh-P4HA2 silencing MDA-MB-231 cells (1 × 10^5^ cells in 200 μl medium) were plated on the top of the Transwell filter and incubated in 37°C 5% CO_2_ for 24 h. The invaded cells on the bottom face of the filter were fixed by methanol and stained with 8% crystal violet. Images were taken with a Nikon microscope and the number of invaded cells was counted.

### Xenograft experiment

Female SCID mice (6 weeks old) were randomly grouped and injected with 2 × 10^6^ sh-control or shP4HA2-1 MDA-MB-231/Luc cells at mammary fat pad. Tumor volume was measured using an *in vivo* imaging system (IVIS). Tumors were measured with a caliper every 4 days for 6 weeks. At the experimental endpoint, tumors were harvested and fixed with 4% PFA for paraffin-embedded section. All procedures were performed within the guidelines of the Division of Laboratory Animal Resources at the University of Kentucky.

### Lung metastasis experiment

Female SCID mice (6 weeks old) were randomly grouped and injected with 1 × 10^6^ (in 200 μl PBS) sh-control or sh-P4HA2-1 MDA-MB-231/Luc cells via tail vein. To detect lung metastasis, bioluminescent images were taken day 30 after cancer cells injection with IVIS Spectrum. Mice were sacrificed week 5 after cancer cells injection.

### Kaplan Meier survival analysis and other statistical analyses

Kaplan-Meier survival analysis was performed in a large combined breast cancer dataset [34]. Breast cancer patients were grouped by estrogen receptor (ER)-positive (n = 1452) and ER-negative (n = 473), and tumor samples were equally grouped into low and high P4HA2 expression based on the mRNA levels. Significant differences in overall survival time were assessed with the Cox proportional hazard (log-rank) test.

Analysis of *P4HA2* mRNA levels in normal and malignant tissues was performed in the TCGA breast cancer dataset that was downloaded from Oncomine. The association between mRNA levels of P4HA2 and collagen genes was evaluated by the Spearman correlation analysis. All experiments were repeated at least twice. Results are reported as mean ± S.E.M; the significance of difference was assessed by independent Student’s t-test. P < 0.05 represents statistical significance and P < 0.01 represents sufficiently statistical significance. All reported P values were 2-tailed. Statistical analysis was conducted with SigmaPlot (Systat Software, Inc.) and SAS (version 9.2; SAS Institute Inc.).

## Results and discussion

### P4HA2 is associated with collagen expression and poor prognosis in human breast cancer

**Figure 1:**
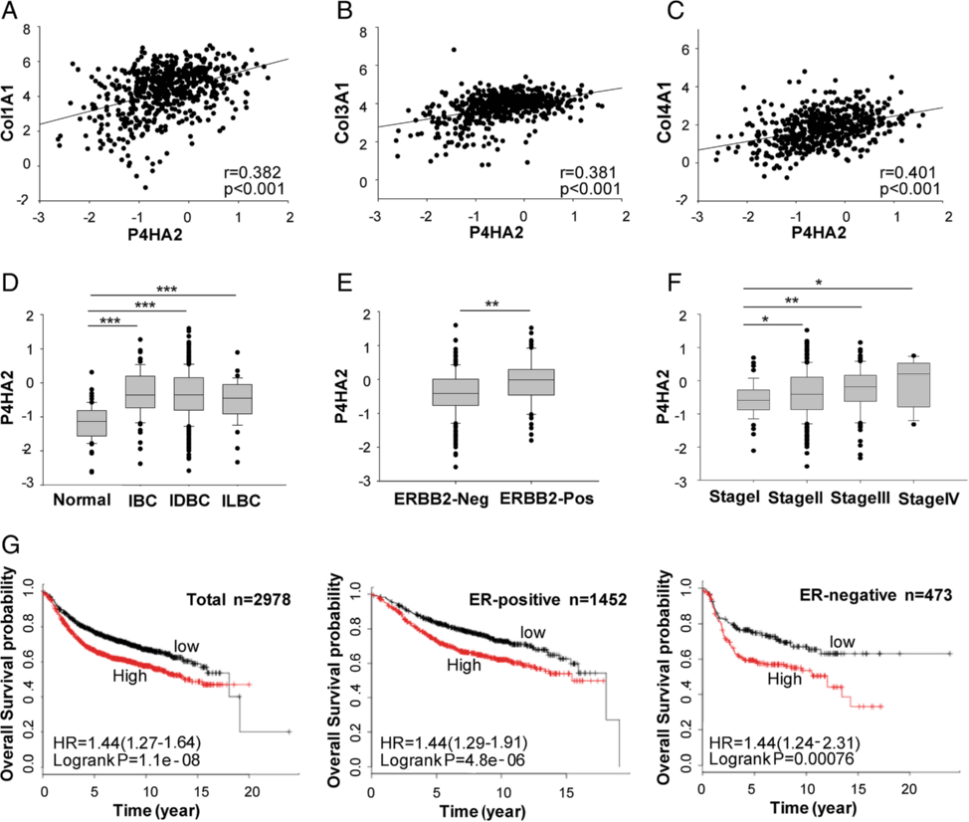
**P4HA2 is associated with collagen expression and poor prognosis in human breast cancer. (A-C)** Scatterplot of correlated mRNA levels between *P4HA2* and **(A)***Col1A1*, **(B)***Col3A1,* and **(C)***Col4A1* in normal and malignant breast tissues (n = 593). The mRNA levels of *P4HA2* were acquired from the TCGA microarray dataset generated from human breast cancer. **(D)***P4HA2* mRNA levels in IBC (invasive breast carcinoma) (n = 76), IDBC (invasive ductal breast carcinoma) (n = 398) and ILBC (invasive lobular breast carcinoma) (n = 36) is higher than normal breast tissue (n = 61). **(E)***P4HA2* mRNA expression is higher in ERBB2 (epidermal growth factor receptor 2) negative tumors than in ERBB2-positive cancer tissues. **(F)***P4HA2* mRNA levels are associated with stages of breast cancer. **(G)** Kaplan-Meier survival analysis showed the association between *P4HA2* expression and clinical outcomes. Breast cancer patients were grouped by ER status (ER-positive, n = 1452; ER-negative, n = 473). Tumor samples were classified into low and high *P4HA2* expression based on the mRNA levels. Tumor had high *P4HA2* expression levels in a shorter overall survival period. The association of *P4HA2* expression and clinical outcome is ER status independent (*p < 0.05; **p < 0.01; ***p < 0.001).

To determine which genes contribute to collagen deposition during cancer development and progression, we performed gene co-expression analysis using the published microarray datasets generated from human breast cancer tissues [35]. *P4HA2* was identified as one of the genes associated with ECM protein expression [35]. By analyzing gene expression in the TCGA breast cancer dataset downloaded from Oncomine, we found expression of *Col1A1*, *Col3A1*, and *Col4A1* was significantly correlated with *P4HA2* levels in normal and malignant breast tissues (p < 0.001, Figure 1A, B and C). Similar results were also obtained in another published microarray dataset [36] (Additional file 1: Figure S1). To assess whether *P4HA2* expression is associated with breast cancer development and progression, we analyzed mRNA levels of *P4HA2* in the TCGA human breast cancer microarray dataset. *P4HA2* expression was significantly upregulated in invasive breast carcinoma (p < 0.001), invasive ductal breast carcinoma (p < 0.001) and invasive lobular breast carcinoma (p < 0.001) compared to normal breast tissue (Figure 1D). We also analyzed the association of *P4HA2* with a number of molecular markers of breast cancer, such as ERBB2 (epidermal growth factor receptor 2), ER, and PR. *P4HA2* expression was significantly upregulated in ERBB2-positive breast cancers compared to ERBB2-negative breast cancers (p < 0.01) (Figure 1E), but *P4HA2* levels had no significant difference between ER- or PR-positive and negative cancer (data not shown). These data confirm the previous finding that *P4HA2* is associated with ERBB2 in human breast cancer cells [29]. We also found that high stage breast cancer had significantly increased P4HA2 expression (Figure 1F). These results indicate that breast cancer development and progression is accompanied by activation P4HA2, which may contribute to collagen synthesis.

A number of genes encoding collagen proteins have been identified as prognostic markers for human breast cancer [37,38]. Since expression of *P4HA2* and collagen genes is correlated in human breast cancer tissues, we asked whether *P4HA2* expression is associated with clinical outcome in human breast cancer patients. Breast cancer patients were divided into two groups based on *P4HA2* mRNA levels (low and high). Kaplan-Meier log rank analysis showed that patients whose tumors had high *P4HA2* expression levels had a significantly shorter overall survival period (Figure 1G). Moreover, the association of *P4HA2* with clinical outcome is ER status independent (see Figure 1G).

#### Inhibition of P4HA2 suppresses the malignant phenotypes of breast cancer cells in 3D culture

**Figure 2:**
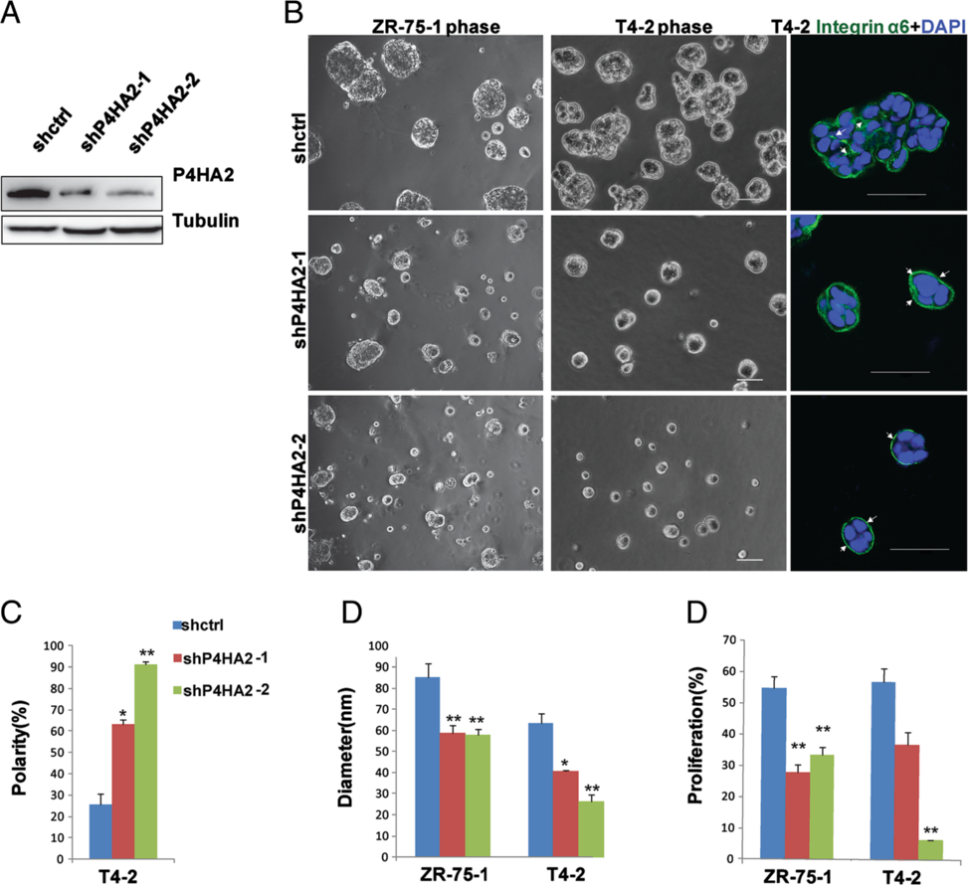
**Silencing P4HA2 reduces colony size and proliferation of breast cancer cells in 3D culture. (A)** P4HA2 knockdown efficiency in T4-2 cells was verified by Western blot. **(B)** Phase images of control shRNA (shctrl) and shP4HA2-expressing ZR-75-1 and T4-2 cells in 3D culture for 4 days. The right column shows immunofluorescence images of α6-integrin (green) and DAPI (blue) staining. P4HA2-silenced T4-2 cells formed polarized acinar structures (arrows pointing to basal surface staining of α6-integrin), whereas control T4-2 cells still maintained disorganized mass-like structures (arrows pointing to lateral staining of α6-integrin; scale bar, 50 μm). **(C)** Ratio of polarized colony in control and knockdown P4HA2 T4-2 cells in 3D culture. **(D)** Quantification of colony size of control and P4HA2-silenced ZR-75-1 and T4-2 cells in 3D culture by measuring the diameter of at least 50 colonies. Knockdown of P4HA2 decreased the colony size. **(E)** EdU-staining was used to analyze the proliferation of control and P4HA2-silenced ZR-75-1 and T4-2 cells in 3D culture. Knockdown P4HA2 inhibited the proliferation of ZR-75-1 and T4-2 cells (*p < 0.05; **p < 0.01).

Increased expression of P4HA2 has been detected in many cancers [27-30], but roles of P4HA2 in cancer progression remain to be determined. To examine the function of P4HA2 in breast cancer progression, we silenced P4HA2 expression in a panel of breast cancer cell lines (HMT-3522 T4-2, MDA-MB-231, ZR-75-1, and MDA-MB-157) with two different shRNAs (shP4HA2-1 and shP4HA2-2). The P4HA2 knockdown efficiency in T4-2 cells was examined by Western blotting (Figure 2A). Similar knockdown efficiency was also obtained in MDA-MB-231 and ZR-75-1 cells (Additional file 1: Figure S2). 3D culture models have been widely used to examine the malignant mammary tissue morphogenesis [31], and the specific 3D phenotypes of breast cancer cells are associated with tumor development and cancer invasiveness. The breast cancer cell lines were classified into four groups based on their phenotypes in 3D culture: round, mass, grape-like, and stellate [39]. Malignant T4-2 cells usually form mass-like structures without apical-basal polarity in Matrigel. Disruption of polarized acinar structure is an early cellular event of tumor development, and 3D culture of T4-2 cells has been used to monitor this process. Knockdown of P4HA2 reprogrammed T4-2 cells to form polarized spheroid structures with reduced colony size (Figure 2B, C, D). Integrin α6 subunit has been used a basal marker to detect basal polarity in mammary epithelial cells [40,41]. Immunofluorescence staining of α6 integrin showed that knockdown of P4HA2 reprogrammed the T4-2 cells to form polarized acinar-like structures (basal staining of α6 integrin), while the control cells formed the unpolarized (lateral staining of α6 integrin) and mass-like morphology (Figure 2B, C). Since disruption of polarized acinar structure is an early event during breast cancer development, these results suggest that P4HA2 contributes to the early stage of breast cancer progression. Knockdown of P4HA2 in T4-2 and ZR-75-1 cells also significantly reduced colony size in 3D culture (Figure 2B, D). To determine whether reduced colony size is due to growth inhibition, cell proliferation was examined by an EdU (5-ethynyl-2′-deoxyuridine) labeling assay as described previously [42,43]. We found that EdU positive cells were significantly reduced in P4HA2-silenced ZR-75-1 and T4-2 cells compared to control cells (Figure 2E).

**Figure 3:**
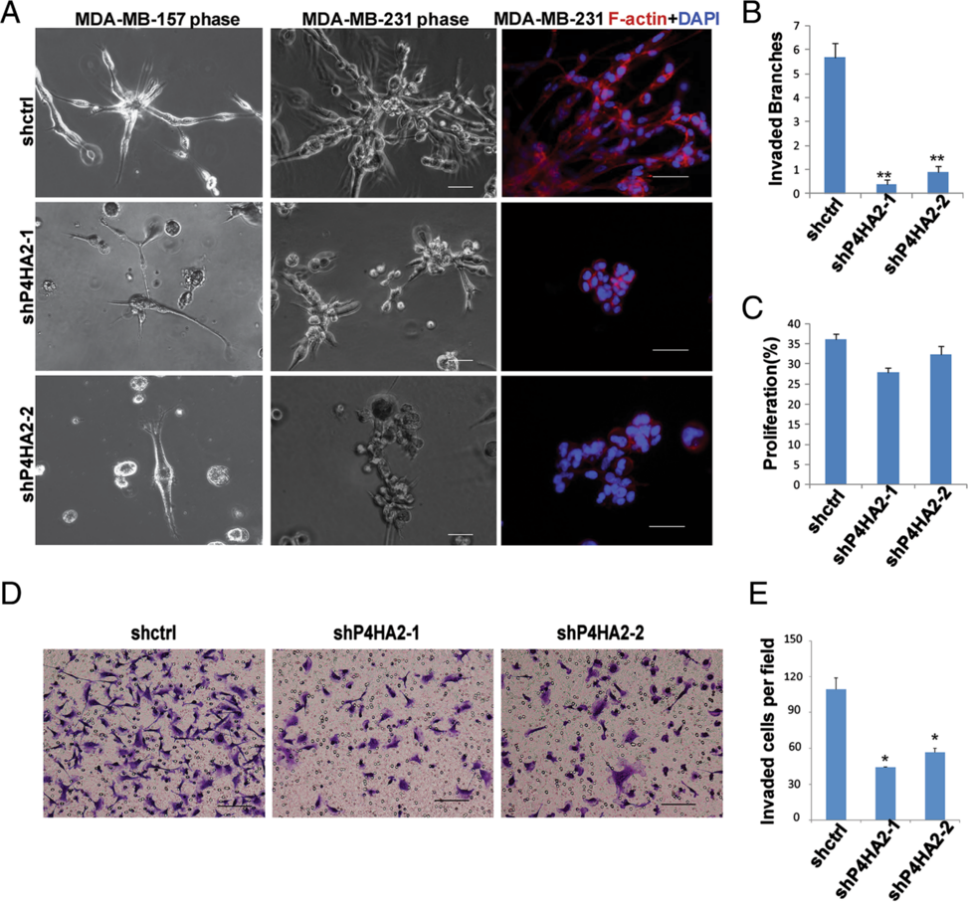
**Knockdown of P4HA2 suppresses breast cancer cells invasiveness in 3D culture. (A)** Phase images of control and P4HA2-silenced MDA-MB-157 and MDA-MB-231 cells in 3D culture for 4 days. The right column shows immunofluorescence images of F-actin (red) and DAPI (blue) staining. P4HA2-silenced MDA-MB-157 and MDA-MB-231 cells formed smaller and less invasive cell clusters than control cells (scale bar, 50 μm). **(B)** Quantification of the invasive branches of control and P4HA2-silenced MDA-MB-157 and MDA-MB-231 cells in 3D culture by counting the branches in at least 50 colonies. Knockdown of P4HA2 reduced the invasive branches. **(C)** EdU-staining was used to analyze the proliferation of control and P4HA2 knockdown MDA-MB-231 cells in 3D culture. Knockdown of P4HA2 had little effect on the proliferation of MDA-MB-231 cells (**p < 0.01). **(D)** Transwell cell invasion assay analysis of control and P4HA2-silenced MDA-MB-231 cells (scale bar, 200 μm). **(E)** Quantification of invasion analysis of control and P4HA2-silenced MDA-MB-231 cells. Knockdown P4HA2 significantly inhibited cell invasion in MDA-MB-231 cells compared with control group.

MDA-MB-231 and MDA-MB-157 cells form stellate structures in 3D culture, which reflects decreased cell-cell interactions and enhanced cell invasiveness [39]. Silencing P4HA2 in MDA-MB-231 cells and MDA-MB-157 cells significantly reduced invasive branches compared to the control cells (Figure 3A, B). P4HA2-silenced cells also had significantly reduced cell invasion in the Transwell assay (Figure 3D, E). Surprisingly, knockdown of P4HA2 had little effect on cell proliferation in MDA-MB-231 in 3D culture system (Figure 3C). Thus, P4HA2 activity in regulating cell proliferation may be cancer stage- and/or subtype-dependent. Nevertheless, reduced invasive branches in P4HA2-silenced cells indicate that P4HA2 contributes to malignant tissue morphogenesis and cancer cell invasion in 3D culture.

**Figure 4:**
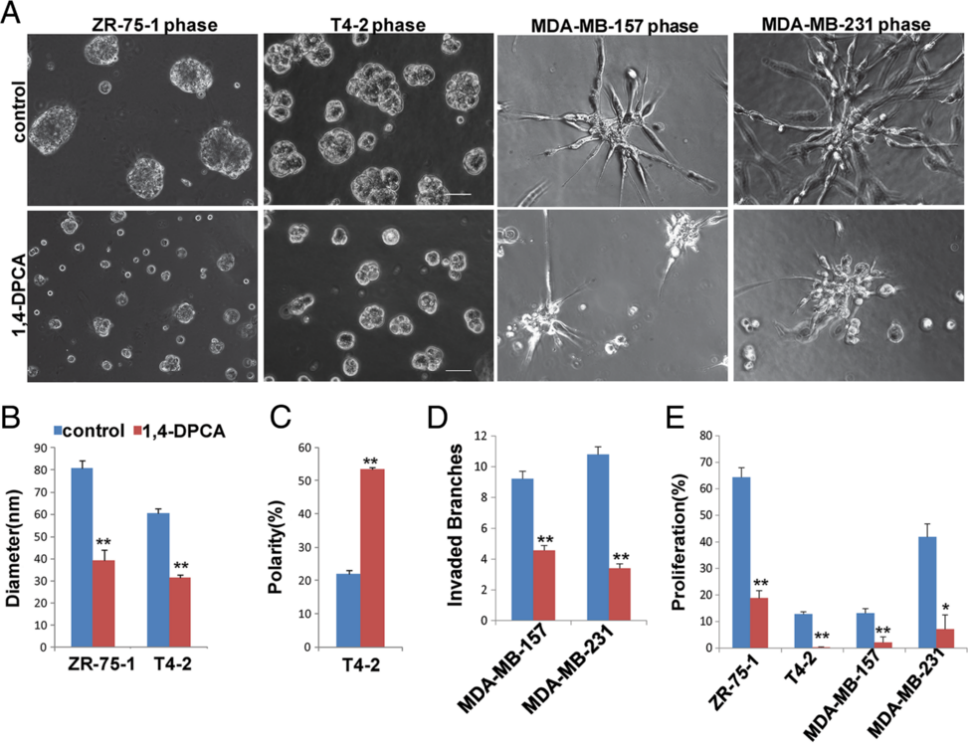
**Treatment with P4HA inhibitor attenuates breast cancer cell proliferation and invasiveness. (A)** Phase images of control and 1,4-DPCA treated ZR-75-1 (20 μM 1,4-DPCA), T4-2 (10 μM 1,4-DPCA), MDA-MB-157 (20 μM 1,4-DPCA) and MDA-MB-231 (10 μM 1,4-DPCA) cells in 3D culture. The cells treated with 1,4-DPCA formed smaller and less aggressive structures compared to control cells (scale bar, 50 μm). **(B)** Quantification of colony size of control and 1,4-DPCA treated ZR-75-1 and T4-2 cells in 3D culture by measuring the diameter of at least 50 colonies. Treatment with 1,4-DPCA reduced the colony size. **(C)** Ratio of polarized colonies in control and 1,4-DPCA treated T4-2 cells in 3D culture. **(D)** Quantification of the invaded branches of control and the 1,4-DPCA treated MDA-MB-157 and MDA-MB-231 cells in 3D culture by measuring the branches in at least 50 colonies. Cells treated with 1,4-DPCA had decreased invasive branch number. **(E)** EdU-staining was used to analyze the proliferation of control and the 1,4-DPCA treated ZR-75-1, T4-2, MDA-MB-157 and MDA-MB-231 cells in 3D culture. Treatment with 1,4-DPCA decreased proliferation of these four cell lines (*p < 0.05; **p < 0.01).

1,4 dihydrophenonthrolin-4-one-3-carboxylic acid (1,4-DPCA) has been identified as a high efficiency inhibitor of prolyl-4-hydroxylase [44-46]. To determine whether P4HA2 is a potential therapeutic target for breast cancer, we treated breast cancer cells with 1,4-DPCA in 3D culture. 1,4-DPCA treatment significantly reduced the colony sizes of T4-2 and ZR-75-1 cells (Figure 4A, B). Immunofluorescence staining results showed that 1,4-DPCA-treated T4-2 cells form polarized spheroids in 3D culture (Figure 4C). Treatment with 1,4-DPCA significantly reduced invasive branches in MDA-MB-231 and MDA-MB-157 cells (Figure 4A, D). Additionally, proliferation of T4-2, ZR-75-1, MDA-MB-157 and MDA-MB-231 cells was all inhibited by 1,4-DPCA (Figure 4E). 1,4-DPCA is not a P4HA2-specific inhibitor, and it also inhibits activity of P4HA1 and P4HA3. A recent study shows that P4HA1 and P4HA3 also contribute to breast cancer progression [33]. Therefore, this small molecule may suppress the malignant phenotypes of breast cancer cells in 3D culture by inhibiting all three P4HA isoforms. Further investigation of 1,4-DPCA activity with an *in vivo* mammary tumor model may lead to discovery of a new drug to inhibit breast cancer development and/or progression.

Daniele M. Gilkes *et al.* reported that knockdown of P4HA2 or treatment MDA-MB-231 cells with hydroxylase inhibitor DHB inhibits tumor growth *in vivo*, but little inhibitory effect on cell proliferation was detected in 2D culture assay [33]. 3D culture has been considered a better model for testing drugs and investigating cancer biology compared to 2D culture, and different drug responses between these two culture systems have recently been reported [47-49]. For example, MDA-MB-231 cells in 3D culture are more sensitive to MEK inhibition compared to cells in 2D culture [47]. Using the 3D culture model, we showed that reducing P4HA2 expression or inhibiting its activity significantly inhibited cell proliferation and suppressed the malignant phenotypes in multiple breast cancer cell lines.

### Reducing P4HA2 expression or inhibiting its activity impairs deposition of collagen I and IV

**Figure 5:**
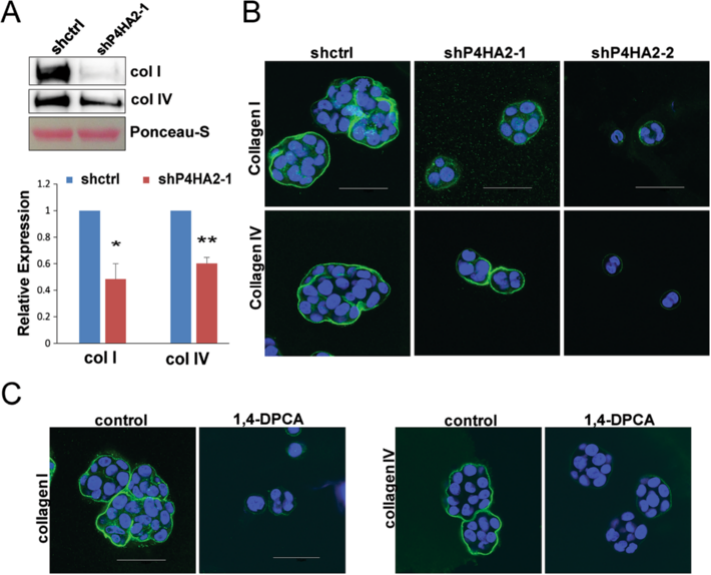
**Reducing P4HA2 expression or inhibiting its activity impairs collagen deposition. (A)** Western blot analysis of collagen I and IV in conditional media generated from the same amount of control and P4HA2-silenced (sh-P4HA2-1) T4-2 cells; bar graph shows quantification of Western blot results (*p < 0.05; **p < 0.01) **(B)** Immunofluorescence staining of collagen I (green) and IV (green) in control and P4HA2-silenced T4-2 cells. P4HA2 knockdown (sh-P4HA2-1) T4-2 cells showed impaired collagen I and IV protein deposition. **(C)** Immunofluorescence staining of collagen I (green) and IV (green) in control and 1,4-DPAC treated T4-2 cells in 3D culture. T4-2 cells treated with 1,4-DPAC showed lower expression of collagen I and IV (scale bar, 50 μm).

Since increased collagen deposition promotes tumor progression by modulating tumor growth and invasion, we asked whether P4HA2 regulates 3D malignant phenotypes of breast cancer cells through enhancing collagen expression and deposition. The conditioned medium was collected from the control and P4HA2-silenced cells (sh-P4HA2-1). Western blot analysis showed that knockdown of P4HA2 in T4-2 cells reduced the protein levels of collagen I and IV in the condition medium (Figure 5A). Deposition of collagen I and IV was also reduced in the P4HA2-silenced and 1,4-DPCA-treated T4-2 cells compared to the control T4-2 cell in 3D culture (Figure 5B, C). These results indicate P4HA2 is crucial for secretion and deposition of collagen I and IV in T4-2 cells. Cancer cells produce a significant amount of ECM proteins and remodeling enzymes [13,50-53]. Dr. Massague’s group recently demonstrated that tenacin-c produced by MDA-MB-231 cells enhances dissemination and survival of tumor cells during the early steps of metastasis by generating a metastatic niche [52]. In addition, breast cancer cells express lysyl oxidases (LOXs), which promote cell invasion by increasing tissue tension and ECM rigidity [13,53]. These results indicate that the ECM microenvironment remodeled by cancer cells is critical for cancer progression. Thus, both inhibiting P4HA2 activity and reducing its expression are potential strategies to suppress collagen-dependent cancer progression.

### P4HA2 regulates tumor growth and metastasis in vivo

**Figure 6:**
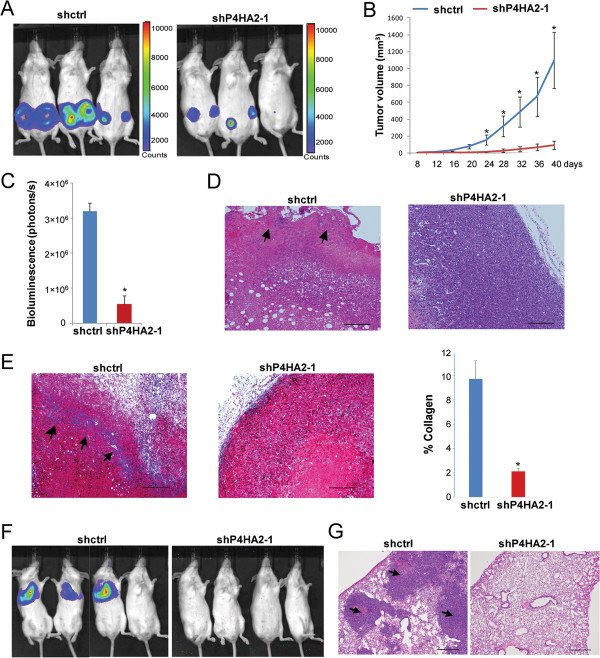
**Knockdown of P4HA2 suppresses tumor growth and metastasis *in vivo*. (A)** Control or P4HA2-silenced (sh-P4HA2-1) MDA-MB-231/Luciferase cells were injected into the mammary fat pad in SCID mice. IVIS images show representative mice from each group (n = 6). **(B)** Tumor growth curve shows that knockdown of P4HA2 (sh-P4HA2-1) inhibited tumor growth in SCID mice (n = 6). **(C)** Tumor volume formed by p4HA2 knockdown (sh-P4HA2-1) cells was significantly reduced compared with control MDA-MB-231/Luc cells. Tumor volume was obtained by quantifying IVIS images. **(D)** Tumor sections were stained with hematoxylin and eosin. Arrows point to invasion area at primary tumor margins (scale bar, 200 μm). **(E)** Masson’s Trichrome staining of tumor sections (blue, collagen fibers; black, nuclei; red, cytoplasm). A significant amount of collagen fibers (arrows) were detected in the control tumors, but not in the P4HA2-silenced (sh-P4HA2-1) tumors (scale bar, 200 μm). The right bar graph is the quantification of Masson’s Trichrome staining results. **(F)** Mice received a tail vein injection of control or P4HA2 knockdown (sh-P4HA2-1) MDA-MB-231/Luc cells (n = 5). IVIS image showed lung metastasis of control and P4HA2-silenced (sh-P4HA2-1) MDA-MB-231 cells in SCID mice. Lung metastasis can be detected in control but not in the knockdown P4HA2 group (*p < 0.05). **(G)** Lung colonization was analyzed by hematoxylin and eosin staining. Arrows point to the tumor formed in control group lung tissue (Scale bar, 500 μm).

Our data showed that silencing P4HA2 in breast cancer cells suppressed their malignant phenotypes and inhibited cell proliferation in 3D culture. We also found that P4HA2 expression is positively associated with breast cancer progression, thus we hypothesized that knockdown of P4HA2 reduces tumor growth and metastasis *in vivo*. To test this hypothesis, we performed xenograft tumor experiments using control and P4HA2-silenced MDA-MB-231 cells. Knockdown of P4HA2 (sh-P4HA2-1) significantly inhibited primary tumor growth in SCID mice (Figure 6A, B and C). HE staining showed that control group tumors had aggressive invasion at primary tumor margins, while tumors in the P4HA2-silenced (sh-P4HA2-1) group had no such invasion (Figure 6D). Collagen deposition in the tumors was assessed by Masson’s Trichrome staining. A significant amount of collagen fibers was detected in the tumors and at the tumor invasion margins in the control group compared to the P4HA2-silenced (sh-P4HA2-1) tumors (Figure 6E). It has been shown that increased collagen deposition and/or orientation/alignment of collagen fibers around tumors enhances cancer progression [5,10,12,14]. Cancer cell invasion usually occurs at the tumor-stromal interface with oriented collagen fibers, and aligned collagen fibers can facilitate cell migration and metastasis [5,10,15,54]. These results suggest that P4HA2 promotes tumor growth and invasion through enhancing collagen deposition.

To determine whether P4HA2 promotes breast cancer lung metastasis *in vivo*, the control and P4HA2-silenced (sh-P4HA2-1) MDA-MB-231 cells were injected into the tail veins of SCID mice. Lung colonization of the cancer cells was monitored by IVIS imaging. We showed that the mice injected with control cells developed lung metastasis within 6 weeks, while no metastasis was detected in the P4HA2-silenced group (Figure 6F). HE staining further confirmed that knockdown of P4HA2 blocked the lung colonization of MDA-MB-231 cells in SCID mice (Figure 6G).

## Conclusion

In the present study, we show that P4HA2 is associated with expression of collagen I, III, and IV during breast cancer progression. Increased mRNA levels of P4HA2 correlate with poor prognosis in human breast cancer patients. Silencing P4HA2 or inhibiting its activity suppresses breast cancer progression by reducing tumor growth and metastasis, and this process is accompanied by reduced collagen deposition. During preparation of this manuscript, Daniele M. Gilkes *et al.* reported that hypoxia-inducible factor 1 activates the transcription of P4HA1 and 2 during breast cancer development, and this activation enhances collagen fiber alignment and breast cancer progression [33,55]. Taken together, these findings indicate that P4HA2 is a promising therapeutic target to inhibit ECM-dependent breast cancer progression.

## Abbreviations

P4HA2: Prolyl-4-hydroxylase α subunit 2; ECM: Extracellular matrix; ER: Estrogen receptor; PR: Progesterone receptor; ERBB2: Epidermal growth factor receptor 2, 1,4-DPCA, 1,4-dihydrophenonthrolin-4-one-3-Carboxylic acid; EdU: 5-ethynyl-2′-deoxyuridine.

## Competing interests

The authors declare that they have no conflict of interest.

## Authors’ contributions

GX participated in the functional assays, *in vivo* experiments with shP4HA2 breast cancer cells and drafted the manuscript. LD participated in the functional assays. JZ conducted Western blotting and helped draft the manuscript. PR participated in the animal experiments. RX conceived of the study, supervised its design and coordination, conducted the bioinformatics analysis and wrote the manuscript. All authors read and approved the final manuscript.

## Pre-publication history

The pre-publication history for this paper can be accessed here:

http://www.biomedcentral.com/1471-2407/14/1/prepub

## Supplementary Material

Additional file 1: Figure S1 Scatterplot of correlated mRNA levels between *P4HA2* and **(A)***Col1A1*, **(B)***Col3A1* and **(C)***Col4A1*. Plots indicate the correlation between *P4HA2* and *Col1A1*, *Col3A1*, *Col4A1* expression in malignant breast tissues (n=118). The mRNA levels of *P4HA2* were acquired from the Chin K’s breast cancer dataset [36]. **Figure S2.** Five shP4HAs were tested in MDA-MB-231 cells by Western blot. ShP4HA2-1 and shP4HA2-2 showed best knockdown efficiency. **Figure S3.** Western blotting experiments shown P4HA2 knock out efficiency in shP4HA2-1 and shP4HA2-2 infected ZR-75-1 cells and MDA-MB-157 cells. Click here for file

## References

[B1] Bissell MJ Hines WC Why don’t we get more cancer? A proposed role of the microenvironment in restraining cancer progression Nat Med 2011 17 3 320 329 10.1038/nm.2328 21383745 PMC3569482

[B2] Lu P Weaver VM Werb Z The extracellular matrix: a dynamic niche in cancer progression J Cell Biol 2012 196 4 395 406 10.1083/jcb.201102147 22351925 PMC3283993

[B3] Muschler J Streuli CH Cell-matrix interactions in mammary gland development and breast cancer Cold Spring Harbor Perspect Biol 2010 2 10 a003202 10.1101/cshperspect.a003202 PMC2944360 20702598

[B4] Lochter A Bissell MJ Involvement of extracellular matrix constituents in breast cancer Semin Cancer Biol 1995 6 3 165 173 10.1006/scbi.1995.0017 7495985

[B5] Provenzano PP Eliceiri KW Campbell JM Inman DR White JG Keely PJ Collagen reorganization at the tumor-stromal interface facilitates local invasion BMC Med 2006 4 1 38 10.1186/1741-7015-4-38 17190588 PMC1781458

[B6] Canty EG Kadler KE Procollagen trafficking, processing and fibrillogenesis J Cell Sci 2005 118 Pt 7 1341 1353 15788652 10.1242/jcs.01731

[B7] Pozzi A Wary KK Giancotti FG Gardner HA Integrin alpha1beta1 mediates a unique collagen-dependent proliferation pathway in vivo J Cell Biol 1998 142 2 587 594 10.1083/jcb.142.2.587 9679154 PMC2133043

[B8] Yeh YC Lin HH Tang MJ A tale of two collagen receptors, integrin beta1 and discoidin domain receptor 1, in epithelial cell differentiation Am J Physiol Cell Physiol 2012 303 12 C1207 C1217 10.1152/ajpcell.00253.2012 23015544

[B9] Zhang K Corsa CA Ponik SM Prior JL Piwnica-Worms D Eliceiri KW Keely PJ Longmore GD The collagen receptor discoidin domain receptor 2 stabilizes SNAIL1 to facilitate breast cancer metastasis Nat Cell Biol 2013 15 6 677 687 10.1038/ncb2743 23644467 PMC3794710

[B10] Provenzano PP Inman DR Eliceiri KW Knittel JG Yan L Rueden CT White JG Keely PJ Collagen density promotes mammary tumor initiation and progression BMC Med 2008 6 11 10.1186/1741-7015-6-11 18442412 PMC2386807

[B11] Shields MA Dangi-Garimella S Krantz SB Bentrem DJ Munshi HG Pancreatic cancer cells respond to type I collagen by inducing snail expression to promote membrane type 1 matrix metalloproteinase-dependent collagen invasion J Biol Chem 2011 286 12 10495 10504 10.1074/jbc.M110.195628 21288898 PMC3060503

[B12] Iyengar P Espina V Williams TW Lin Y Berry D Jelicks LA Lee H Temple K Graves R Pollard J Adipocyte-derived collagen VI affects early mammary tumor progression in vivo, demonstrating a critical interaction in the tumor/stroma microenvironment J Clin Invest 2005 115 5 1163 1176 15841211 10.1172/JCI23424 PMC1077173

[B13] Levental KR Yu H Kass L Lakins JN Egeblad M Erler JT Fong SF Csiszar K Giaccia A Weninger W Matrix crosslinking forces tumor progression by enhancing integrin signaling Cell 2009 139 5 891 906 10.1016/j.cell.2009.10.027 19931152 PMC2788004

[B14] Van ‘t Veer LJ Dai H van de Vijver MJ He YD Hart AA Mao M Peterse HL van der Kooy K Marton MJ Witteveen AT Gene expression profiling predicts clinical outcome of breast cancer Nature 2002 415 6871 530 536 10.1038/415530a 11823860

[B15] Condeelis J Segall JE Intravital imaging of cell movement in tumours Nat Rev Cancer 2003 3 12 921 930 10.1038/nrc1231 14737122

[B16] Myllyharju J Prolyl 4-hydroxylases, the key enzymes of collagen biosynthesis Matrix Biol 2003 22 1 15 24 10.1016/S0945-053X(03)00006-4 12714038

[B17] Gorres KL Raines RT Prolyl 4-hydroxylase Crit Rev Biochem Mol Biol 2010 45 2 106 124 10.3109/10409231003627991 20199358 PMC2841224

[B18] Kukkola L Hieta R Kivirikko KI Myllyharju J Identification and characterization of a third human, rat, and mouse collagen prolyl 4-hydroxylase isoenzyme J Biol Chem 2003 278 48 47685 47693 10.1074/jbc.M306806200 14500733

[B19] Pajunen L Jones TA Helaakoski T Pihlajaniemi T Solomon E Sheer D Kivirikko KI Assignment of the gene coding for the alpha-subunit of prolyl 4-hydroxylase to human chromosome region 10q21.3-23.1 Am J Hum Genet 1989 45 6 829 834 2556027 PMC1683466

[B20] Annunen P Helaakoski T Myllyharju J Veijola J Pihlajaniemi T Kivirikko KI Cloning of the human prolyl 4-hydroxylase alpha subunit isoform alpha(II) and characterization of the type II enzyme tetramer: the alpha(I) and alpha(II) subunits do not form a mixed alpha(I)alpha(II)beta2 tetramer J Biol Chem 1997 272 28 17342 17348 10.1074/jbc.272.28.17342 9211872

[B21] Sundberg C Ivarsson M Gerdin B Rubin K Pericytes as collagen-producing cells in excessive dermal scarring Lab Invest 1996 74 2 452 466 8780163

[B22] Stephens EH Grande-Allen KJ Age-related changes in collagen synthesis and turnover in porcine heart valves J Heart Valve Dis 2007 16 6 672 682 18095519

[B23] Bulleid NJ John DC Kadler KE Recombinant expression systems for the production of collagen Biochem Soc Trans 2000 28 4 350 353 10.1042/0300-5127:0280350 10961917

[B24] Nokelainen M Nissi R Kukkola L Helaakoski T Myllyharju J Characterization of the human and mouse genes for the alpha subunit of type II prolyl 4-hydroxylase: identification of a previously unknown alternatively spliced exon and its expression in various tissues Eur J Biochem 2001 268 20 5300 5309 10.1046/j.0014-2956.2001.02464.x 11606192

[B25] Grimmer C Balbus N Lang U Aigner T Cramer T Muller L Swoboda B Pfander D Regulation of type II collagen synthesis during osteoarthritis by prolyl-4-hydroxylases: possible influence of low oxygen levels Am J Pathol 2006 169 2 491 502 10.2353/ajpath.2006.050738 16877351 PMC1698781

[B26] Myllyharju J Schipani E Extracellular matrix genes as hypoxia-inducible targets Cell Tissue Res 2010 339 1 19 29 10.1007/s00441-009-0841-7 19662436 PMC3074490

[B27] Chang KP Yu JS Chien KY Lee CW Liang Y Liao CT Yen TC Lee LY Huang LL Liu SC Identification of PRDX4 and P4HA2 as metastasis-associated proteins in oral cavity squamous cell carcinoma by comparative tissue proteomics of microdissected specimens using iTRAQ technology J Proteome Res 2011 10 11 4935 4947 10.1021/pr200311p 21859152

[B28] Jarzab B Wiench M Fujarewicz K Simek K Jarzab M Oczko-Wojciechowska M Wloch J Czarniecka A Chmielik E Lange D Gene expression profile of papillary thyroid cancer: sources of variability and diagnostic implications Cancer Res 2005 65 4 1587 1597 10.1158/0008-5472.CAN-04-3078 15735049

[B29] Mackay A Jones C Dexter T Silva RL Bulmer K Jones A Simpson P Harris RA Jat PS Neville AM cDNA microarray analysis of genes associated with ERBB2 (HER2/neu) overexpression in human mammary luminal epithelial cells Oncogene 2003 22 17 2680 2688 10.1038/sj.onc.1206349 12730682

[B30] Pan PW Zhang Q Bai F Hou J Bai G Profiling and comparative analysis of glycoproteins in Hs578BST and Hs578T and investigation of prolyl 4-hydroxylase alpha polypeptide II expression and influence in breast cancer cells Biochemistry (Mosc) 2012 77 5 539 545 10.1134/S000629791205015X 22813596

[B31] Petersen OW Ronnov-Jessen L Howlett AR Bissell MJ Interaction with basement membrane serves to rapidly distinguish growth and differentiation pattern of normal and malignant human breast epithelial cells Proc Natl Acad Sci USA 1992 89 19 9064 9068 10.1073/pnas.89.19.9064 1384042 PMC50065

[B32] Xu R Nelson CM Muschler JL Veiseh M Vonderhaar BK Bissell MJ Sustained activation of STAT5 is essential for chromatin remodeling and maintenance of mammary-specific function J Cell Biol 2009 184 1 57 66 10.1083/jcb.200807021 19139262 PMC2615090

[B33] Gilkes DM Chaturvedi P Bajpai S Wong CC Wei H Pitcairn S Hubbi ME Wirtz D Semenza GL Collagen prolyl hydroxylases are essential for breast cancer metastasis Cancer Res 2013 73 11 3285 3296 10.1158/0008-5472.CAN-12-3963 23539444 PMC3674184

[B34] Gyorffy B Lanczky A Eklund AC Denkert C Budczies J Li Q Szallasi Z An online survival analysis tool to rapidly assess the effect of 22,277 genes on breast cancer prognosis using microarray data of 1,809 patients Breast Cancer Res Treat 2010 123 3 725 731 10.1007/s10549-009-0674-9 20020197

[B35] Xu R Mao JH Gene transcriptional networks integrate microenvironmental signals in human breast cancer Integr Biol: Quant Biosci Nano macro 2011 3 4 368 374 10.1039/c0ib00087f PMC3121540 21165486

[B36] Chin K DeVries S Fridlyand J Spellman PT Roydasgupta R Kuo WL Lapuk A Neve RM Qian Z Ryder T Genomic and transcriptional aberrations linked to breast cancer pathophysiologies Cancer Cell 2006 10 6 529 541 10.1016/j.ccr.2006.10.009 17157792

[B37] Helleman J Jansen MP Ruigrok-Ritstier K van Staveren IL Look MP Meijer-van Gelder ME Sieuwerts AM Klijn JG Sleijfer S Foekens JA Association of an extracellular matrix gene cluster with breast cancer prognosis and endocrine therapy response Clin Cancer Res 2008 14 17 5555 5564 10.1158/1078-0432.CCR-08-0555 18765548

[B38] Wang Y Klijn JG Zhang Y Sieuwerts AM Look MP Yang F Talantov D Timmermans M Meijer-van Gelder ME Yu J Gene-expression profiles to predict distant metastasis of lymph-node-negative primary breast cancer Lancet 2005 365 9460 671 679 15721472 10.1016/S0140-6736(05)17947-1

[B39] Kenny PA Lee GY Myers CA Neve RM Semeiks JR Spellman PT Lorenz K Lee EH Barcellos-Hoff MH Petersen OW The morphologies of breast cancer cell lines in three-dimensional assays correlate with their profiles of gene expression Mol Oncol 2007 1 1 84 96 10.1016/j.molonc.2007.02.004 18516279 PMC2391005

[B40] Wang F Weaver VM Petersen OW Larabell CA Dedhar S Briand P Lupu R Bissell MJ Reciprocal interactions between beta1-integrin and epidermal growth factor receptor in three-dimensional basement membrane breast cultures: a different perspective in epithelial biology Proc Natl Acad Sci USA 1998 95 25 14821 14826 10.1073/pnas.95.25.14821 9843973 PMC24533

[B41] Weaver VM Petersen OW Wang F Larabell CA Briand P Damsky C Bissell MJ Reversion of the malignant phenotype of human breast cells in three-dimensional culture and in vivo by integrin blocking antibodies J Cell Biol 1997 137 1 231 245 10.1083/jcb.137.1.231 9105051 PMC2139858

[B42] Salic A Mitchison TJ A chemical method for fast and sensitive detection of DNA synthesis in vivo Proc Natl Acad Sci USA 2008 105 7 2415 2420 10.1073/pnas.0712168105 18272492 PMC2268151

[B43] Xiong G Wang C Evers BM Zhou BP Xu R RORalpha suppresses breast tumor invasion by inducing SEMA3F expression Cancer Res 2012 72 7 1728 1739 10.1158/0008-5472.CAN-11-2762 22350413 PMC3319846

[B44] Franklin TJ Morris WP Edwards PN Large MS Stephenson R Inhibition of prolyl 4-hydroxylase in vitro and in vivo by members of a novel series of phenanthrolinones Biochem J 2001 353 Pt 2 333 338 11139398 10.1042/0264-6021:3530333 PMC1221576

[B45] Martinez-Outschoorn UE Trimmer C Lin Z Whitaker-Menezes D Chiavarina B Zhou J Wang C Pavlides S Martinez-Cantarin MP Capozza F Autophagy in cancer associated fibroblasts promotes tumor cell survival: Role of hypoxia, HIF1 induction and NFkappaB activation in the tumor stromal microenvironment Cell Cycle 2010 9 17 3515 3533 10.4161/cc.9.17.12928 20855962 PMC3047617

[B46] Sondo E Tomati V Caci E Esposito AI Pfeffer U Pedemonte N Galietta LJ Rescue of the mutant CFTR chloride channel by pharmacological correctors and low temperature analyzed by gene expression profiling Am J Physiol Cell Physiol 2011 301 4 C872 C885 10.1152/ajpcell.00507.2010 21753184 PMC3512166

[B47] Li Q Chow AB Mattingly RR Three-dimensional overlay culture models of human breast cancer reveal a critical sensitivity to mitogen-activated protein kinase kinase inhibitors J Pharmacol Exper Ther 2010 332 3 821 828 10.1124/jpet.109.160390 19952304 PMC2835447

[B48] Muranen T Selfors LM Worster DT Iwanicki MP Song L Morales FC Gao S Mills GB Brugge JS Inhibition of PI3K/mTOR leads to adaptive resistance in matrix-attached cancer cells Cancer Cell 2012 21 2 227 239 10.1016/j.ccr.2011.12.024 22340595 PMC3297962

[B49] Pickl M Ries CH Comparison of 3D and 2D tumor models reveals enhanced HER2 activation in 3D associated with an increased response to trastuzumab Oncogene 2009 28 3 461 468 10.1038/onc.2008.394 18978815

[B50] Spencer VA Xu R Bissell MJ Extracellular matrix, nuclear and chromatin structure, and gene expression in normal tissues and malignant tumors: a work in progress Adv Cancer Res 2007 97 275 294 17419950 10.1016/S0065-230X(06)97012-2 PMC2912285

[B51] Calvo F Ege N Grande-Garcia A Hooper S Jenkins RP Chaudhry SI Harrington K Williamson P Moeendarbary E Charras G Mechanotransduction and YAP-dependent matrix remodelling is required for the generation and maintenance of cancer-associated fibroblasts Nat Cell Biol 2013 15 6 637 646 10.1038/ncb2756 23708000 PMC3836234

[B52] Oskarsson T Acharyya S Zhang XH Vanharanta S Tavazoie SF Morris PG Downey RJ Manova-Todorova K Brogi E Massague J Breast cancer cells produce tenascin C as a metastatic niche component to colonize the lungs Nat Med 2011 17 7 867 874 10.1038/nm.2379 21706029 PMC4020577

[B53] El-Haibi CP Bell GW Zhang J Collmann AY Wood D Scherber CM Csizmadia E Mariani O Zhu C Campagne A Critical role for lysyl oxidase in mesenchymal stem cell-driven breast cancer malignancy Proc Natl Acad Sci USA 2012 109 43 17460 17465 10.1073/pnas.1206653109 23033492 PMC3491529

[B54] Brownfield DG Venugopalan G Lo A Mori H Tanner K Fletcher DA Bissell MJ Patterned collagen fibers orient branching mammary epithelium through distinct signaling modules Curr Biol 2013 23 8 703 709 10.1016/j.cub.2013.03.032 23562267 PMC3705902

[B55] Gilkes DM Bajpai S Chaturvedi P Wirtz D Semenza GL Hypoxia-inducible factor 1 (HIF-1) promotes extracellular matrix remodeling under hypoxic conditions by inducing P4HA1, P4HA2, and PLOD2 expression in fibroblasts J Biol Chem 2013 288 15 10819 10829 10.1074/jbc.M112.442939 23423382 PMC3624462

