# Supplementary material for: Prolyl-4-hydroxylase α subunit 2 promotes breast cancer progression and metastasis by regulating collagen deposition
Source: BMC Cancer. 2014 Jan 2;14:1. doi: 10.1186/1471-2407-14-1 (PMC3880410; doi:10.1186/1471-2407-14-1)
Supplement: Additional file 1: Figure S1 — Scatterplot of correlated mRNA levels between P4HA2 and (A)Col1A1, (B)Col3A1 and (C)Col4A1. Plots indicate the correlation between P4HA2 and Col1A1, Col3A1, Col4A1 expression in malignant breast tissues (n=118). The mRNA levels of P4HA2 were acquired from the Chin K’s breast cancer dataset [36]. Figure S2. Five shP4HAs were tested in MDA-MB-231 cells by Western blot. ShP4HA2-1 and shP4HA2-2 showed best knockdown efficiency. Figure S3. Western blotting experiments shown P4HA2 knock out efficiency in shP4HA2-1 and shP4HA2-2 infected ZR-75-1 cells and MDA-MB-157 cells. [file 1471-2407-14-1-S1.pdf]

Supplemental Figure 1. Scatterplot of correlated mRNA levels between *P4HA2* and (**A**) *Col1A1*, (**B**) *Col3A1* and (**C**) *Col4A1*. Plots indicate the correlation between *P4HA2* and *Col1A1*, *Col3A1*, *Col4A1* expression in malignant breast tissues (n=118). The mRNA levels of *P4HA2* were acquired from the Chin K's breast cancer dataset [36].

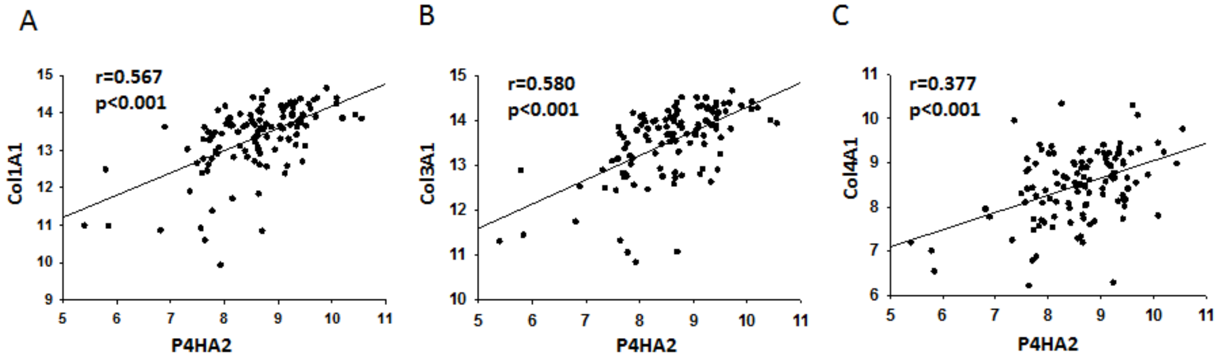

Supplemental Figure 2. Five shP4HAs were tested in MDA-MB-231 cells by Western blot. ShP4HA2-1 and shP4HA2-2 showed best knockdown efficiency.

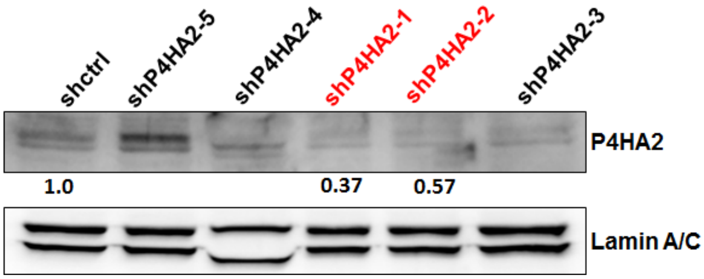

Supplemental Figure 3. Western blotting experiments shown P4HA2 knock out efficiency in shP4HA2-1 and shP4HA2-2 infected ZR-75-1 cells and MDA-MB-157 cells.

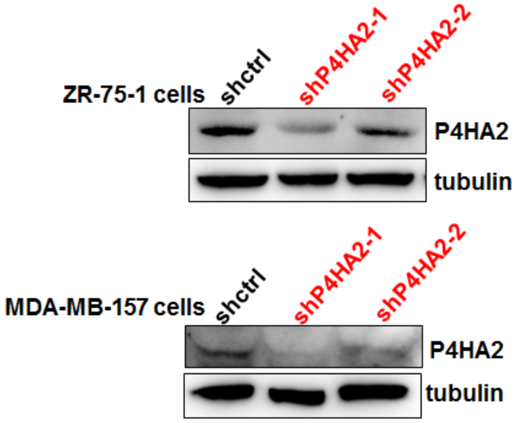

ZR-75-1 cells
shctrl
shP4HA2-1
shP4HA2-2
P4HA2
tubulin
MDA-MB-157 cells
shctrl
shP4HA2-1
shP4HA2-2
P4HA2
tubulin
